# Tensile testing of the mechanical behavior of the human periodontal ligament

**DOI:** 10.1186/s12938-018-0607-0

**Published:** 2018-11-23

**Authors:** Bin Wu, Yipeng Fu, Haotian Shi, Bin Yan, Ruxin Lu, Songyun Ma, Bernd Markert

**Affiliations:** 1 College of Mechanical and Electronic Engineering, Nanjing Forestry University, Nanjing, China; 2 Jiangsu Key Laboratory of Oral Diseases, Nanjing Medical University, Nanjing, China; 3 Department of Orthodontics, Affiliated Hospital of Stomatology, Nanjing Medical University, Nanjing, China; 4 Institute of General Mechanics, RWTH-Aachen University, Aachen, Germany

**Keywords:** Human periodontal ligament, Uniaxial tensile test, Elastic modulus, Biomechanics

## Abstract

**Background:**

The periodontal ligament (PDL) plays a key role in alveolar bone remodeling and resorption during tooth movements. The prediction of tooth mobility under functional dental loads requires a deep understanding of the mechanical behavior of the PDL, which is a critical issue in dental biomechanics. This study was aimed to examine the mechanical behavior of the PDL of the maxillary central and lateral incisors from human. The experimental results can contribute to developing an accurate constitutive model of the human PDL in orthodontics.

**Methods:**

The samples of human incisors were cut into three slices. Uniaxial tensile tests were conducted under different loading rates. The transverse sections (cervical, middle and apex) normal to the longitudinal axis of the root of the tooth were used in the uniaxial tensile tests. Based on a bilinear simplification of the stress–strain relations, the elastic modulus of the PDL was calculated. The values of the elastic modulus in different regions were compared to explore the factors that influence the mechanical behavior of the periodontal ligament.

**Results:**

The obtained stress–strain curves of the human PDL were characterized by a bilinear model with two moduli (E_1_ and E_2_) for quantifying the elastic behavior of the PDL from the central and lateral incisors. Statistically significant differences of the elastic modulus were observed in the cases of 1, 3, and 5 N loading levels for the different teeth (central and lateral incisors). The results showed that the mechanical property of the human incisors’ PDLs is dependent on the location of PDL (ANOVA, *P* = 0.022, *P* < 0.05). The elastic moduli at the middle planes were greater than at the cervical and apical planes. However, at the cervical, middle, and apical planes, the elastic moduli of the mesial and distal site were not significantly different (ANOVA, *P* = 0.804, *P* > 0.05).

**Conclusions:**

The values of elastic modulus were determined in the range between 0.607 and 4.274 MPa under loads ranging from 1 to 5 N. The elastic behavior of the PDL is influenced by the loading rate, tooth type, root level, and individual variation.

## Background

The periodontal ligament (PDL) is a soft, thin layer connective tissue that transmits forces to the alveolar bone. Under orthodontic forces, the thickness of the PDL is decreased, which causes the shift of the tooth. The PDL plays a key role in alveolar bone remodeling and resorption during tooth movements [1]. Accurate prediction of tooth mobility under functional dental loads is an open issue in dental biomechanics. Therefore, a deep understanding of the mechanical behavior of the PDL is required for shedding light on the function of the PDL in tooth movement under prosthodontic and orthodontic treatments [2].

The human PDL is only approximately 0.15–0.38 mm wide and composed of type I collagen fibers (53–74%) and the surrounding ground substance matrix [3]. Komatsu [4] observed that the mechanical strength and viscoelastic response of the PDL is related to its structure of the PDL. However, very limited studies have been conducted to examine the relationship between the mechanical properties of the human PDL and the tooth position. The morphological diversity and structural complexity of the PDL lead to a challenge in the investigation of its mechanical properties. Furthermore, it’s hard to separate the PDL from the bone–PDL–tooth complex without cutting the collagen fibers. Biomechanical tests of the PDL have been carried out for decades. Pini [5], Komatsu [6, 7] and Oskui [8] used PDL transverse sections from rabbits, cows, and beagles in the uniaxial tensile tests. However, animal material does not exactly replicate the properties of human tissue. The experimental method using the human PDL transverse sections proposed by Ralph [9] is a useful technique for determining the mechanical properties of the PDL. Mandel [10] measured the whole stress–strain curves, instead of just maximum stress before breaking. In the recent in vitro PDL studies [11, 12], the elastic modulus of PDL varied from 0.01 to 1750 MPa in the uniaxial tests.

In this study, the effects of various factors, including the loading rate, root level and tooth position, on the elastic modulus of the PDL were investigated. The objective of this work was to determine the mechanical behavior of the human PDL experimentally by means of uniaxial tension testing.

## Methods

### Specimen preparation

This study was reviewed and approved (No. (2015)169) by the Institutional Review Board (IRB) of Nanjing Medical University. Maxillary jaw segments from three fresh corpses (male, 31–52 years old, in good dental health, without any periodontal disease) were brought to the laboratory and stored in a refrigerated container at − 20 °C for a subsequent sectioning. They were not immersed in formalin to keep the biomechanical properties. Within 1 week, the maxillary jaw segments were used to fabricate the samples for the testing. Previous studies have shown that freezing and thawing would not affect the mechanical properties of biological tissues [13, 14].

On the day of testing, the maxillary jaws were removed from the freezer. After the peripheral soft tissues were removed from the maxilla, one complete incisor and the surrounding periodontal ligament and alveolar bones were separated from the other teeth using a saw.

**Fig. 1:**
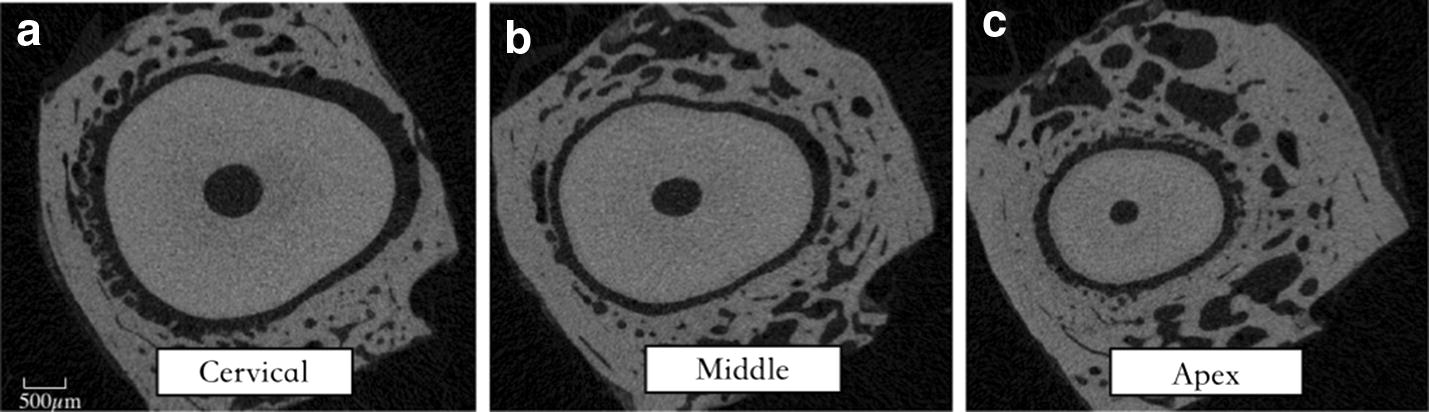
Tooth imaged by micro CT. **a**–**c** Section images of cervical, middle, and apical root level of human PDL

The teeth used for biomechanical testing were imaged [15] by microcomputed tomography (Viva CT 80, Scanco Medical), as shown in Fig. 1. The CT parameter values were set to a 16.1-µm isotropic voxel size, 55 kVp, 145 µA, and 8 W energy. Figure 1a–c show the widths of the PDL at the three-root level (cervical, middle, and apical) in different directions. The Micro CT slices indicate the thickness of the mesial and distal regions are regular, while that of the buccal and palatal regions were inhomogeneous. Therefore, the mesial and distal regions of the PDL were tested.

After the radiographic analysis was completed, each bone–PDL–tooth complex [16] was attached to a fixture half embedded in wax. The complex was sectioned perpendicular to the longitudinal axis of the tooth root using a rotating blade saw [17] (Isomet Low-Speed Saw, Buehler, Lake Bluff, IL, USA) with a speed of 500 r/s. Normal saline was used to keep each specimen cool and prevent heat denaturation. Each transverse section was shaped using portable dental handpiece. The dimensions of each bar-shaped specimen were approximately 8 × 6 × 2 mm. The exact dimensions of each specimen were measured precisely using an electronic digital readout micrometer prior to testing.

**Fig. 2:**
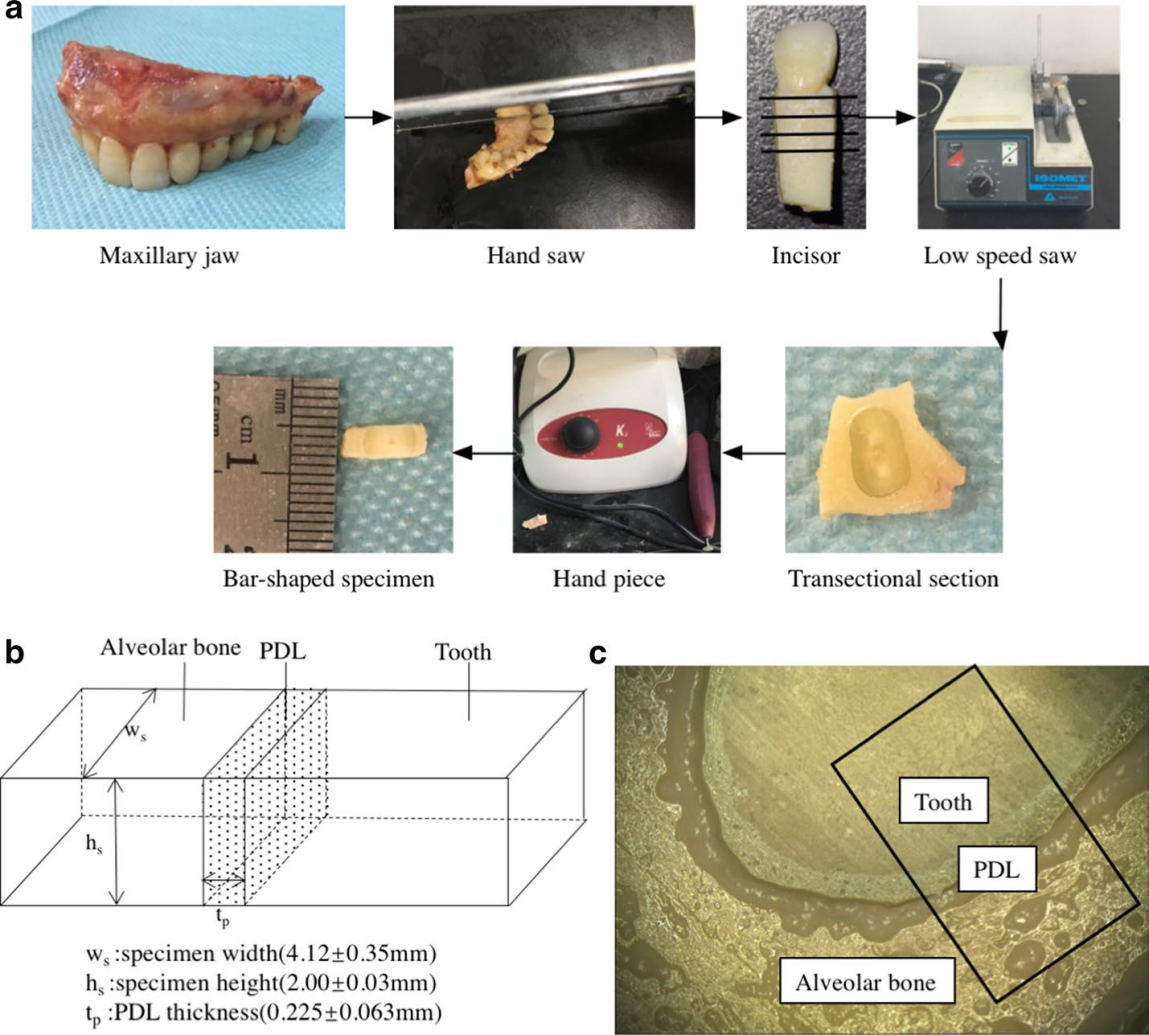
The specimen preparation process. **a** The procedure begins with human maxilla used to form bar-shaped specimens, **b** dimensions of the PDL specimens, **c** photo of bone–PDL–tooth complex under stereomicroscope

The whole process of specimen preparation is illustrated in Fig. 2a. A total of 18 specimens (cervical, middle and apex slices) cut from 6 teeth. 3 central incisors and 3 lateral incisors were prepared. All specimens were shaped from transverse sections, as shown in Fig. 2b, c. The specimens were frozen in normal saline at − 20 °C and stored within a week until they were tested.

### Test procedure

**Fig. 3:**
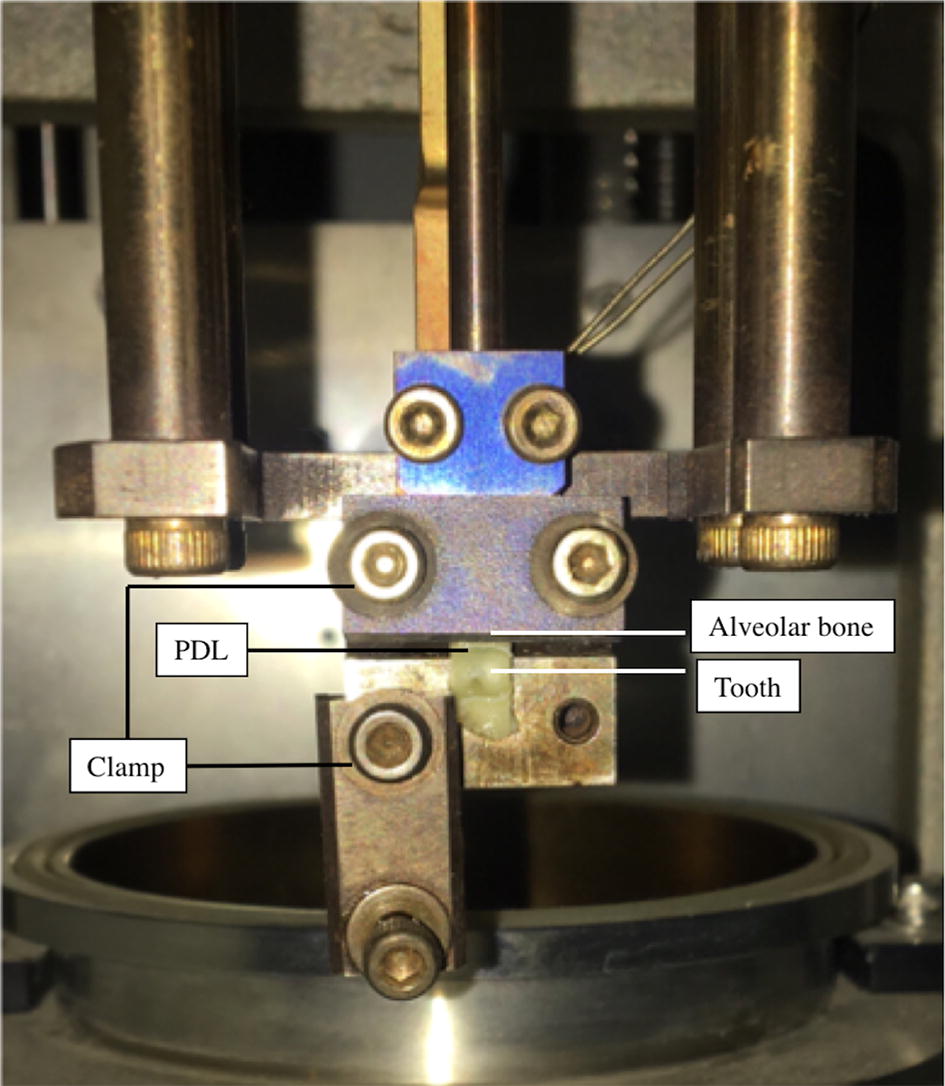
Specimens fixed by the clamps of the DMA equipment

Tensile tests of the bar-shaped PDL specimens were conducted using a Diamond Dynamic Mechanical Analyzer (DMA, PerkinElmer, Inc., Waltham, MA, USA). The alveolar bone and dentin sides of the specimens were fixed using two clamps as illustrated in Fig. 3. To prevent the specimens from drying out and avoid the influence of airflow, the DMA machine’s heat preservation cover was closed during the tests.

#### Preconditioning

Preconditioning of biological tissues before testing is important as it returns the collagen fibers to natural physiological conditions [18]. The majority of earlier studies have used a 1-Hz frequency [19] and displacement amplitude of 35% of the PDL’s initial length [20] in the preconditioning phase. Bergomi [21] stated that 3–5 preconditioning cycles are sufficient to obtain a stable tensile response. In this study, preconditioning was performed on the specimens by applying five harmonic cycles at an amplitude of approximately 10% of the PDL’s initial width to avoid rupture of the PDL. Because the average human chewing frequency is 1.57 Hz [22], the frequency was set to 1 Hz.

#### Uniaxial tensile tests

**Fig. 4:**
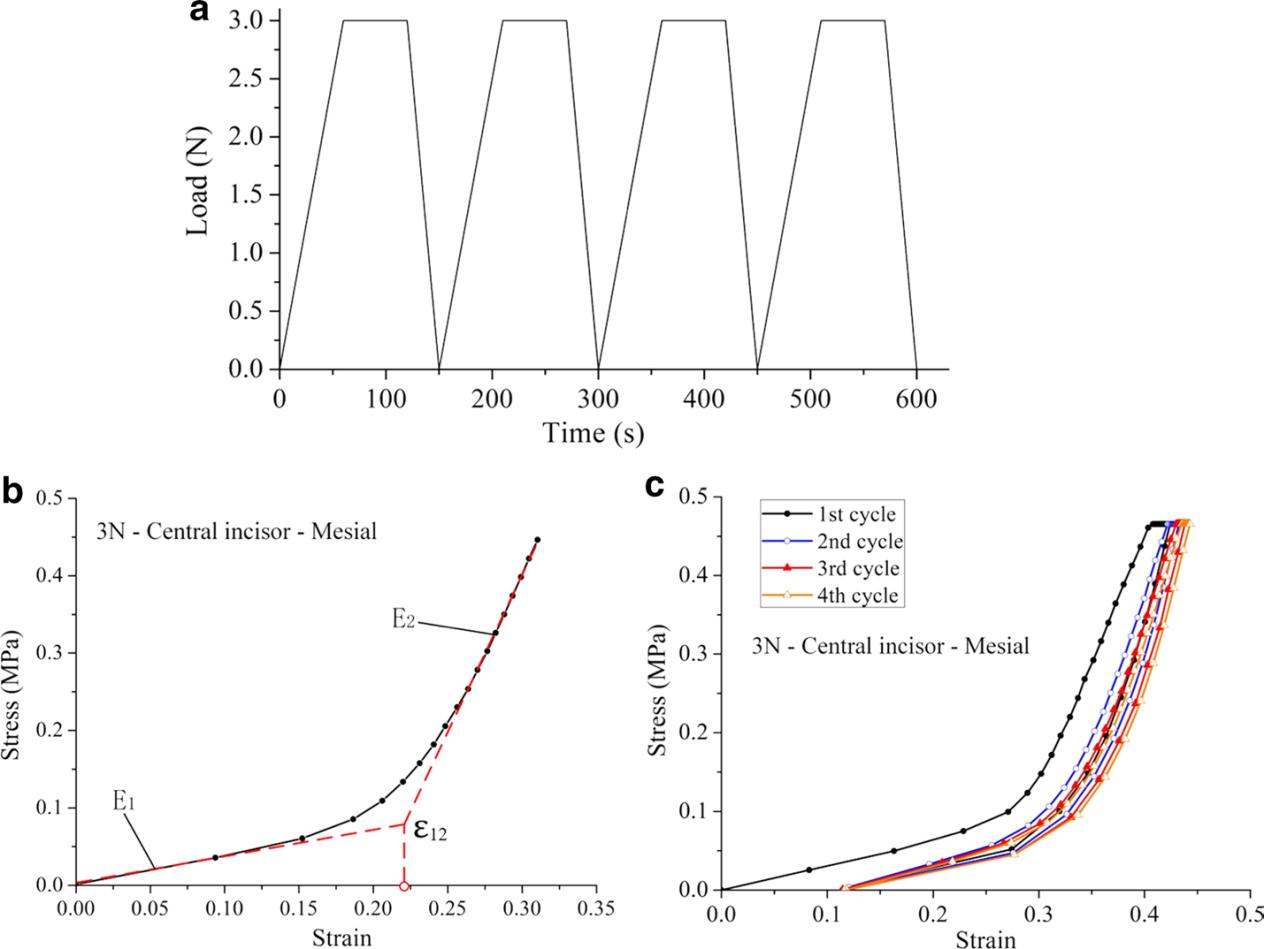
Loading cycles and stress–strain curves of the load case with 3 N force amplitude. **a** Loading cycles in the uniaxial tensile test, **b** illustration of the procedures for determining E_1_, E_2_ and ɛ_12_ of the bilinear elastic model, **c** stress–strain curves of the PDL for four loading cycles

After preconditioning, each specimen was loaded within 1 min (Fig. 4a) to three force levels (1, 3, and 5 N), held for 1 min, and then unloaded. The unloading rate is twice of the loading rate for reducing the testing time and avoiding dry-out of samples according to the suggestion in [23]. The loading-hold-unloading cycle is repeated four times as Fig. 4 in order to perform a statistical analysis of experimental data. The load–displacement curve was recorded by the DMA machine. Between tests, normal saline was sprayed on the specimen to keep it wet [19]. All of the tests were conducted at an indoor air temperature. The parameters of the test procedures are shown in Table 1.

**Table 1:** Parameters of uniaxial tensile tests

Test	Loading rate (N/min)	Maximum load (N)	Hold time (s)	Unloading rate (N/min)	Circle
Uniaxial tension	1	1	60	2	4
	3	3	60	6	4
	5	5	60	10	4

### Stress–strain data analysis

The stress–strain curves were obtained from the experimental load–displacement data. The engineering stress *σ*(*t*) and strain *ɛ*(*t*) at time t were calculated from the load *F*(*t*) and the displacement *D*(*t*), by using the formulae *σ*(*t*) = *F*(*t*)*/A*_0_ and *ɛ*(*t*) = *D*(*t*)*/t*_*p*_, respectively [7]. Here, *A*_0_ = * w*_*s*_ * *h*_*s*_ is the initial area of the transverse section and *t*_*p*_ is the initial width of the specimen, as shown in Fig. 2b. In the previous experimental and computational studies on the PDL [24, 25], the nonlinear stress strain curve of the PDL was approximated by bilinear elastic model. Therefore, we utilize the bilinear model to characterize the mechanical behavior of the human PDL of the maxillary central and lateral incisors. A one-way analysis of variance (ANOVA) was conducted using IBM SPSS Statistics Version 20 (SPSS, Inc., Chicago, IL, USA) for the three sets of experimental data obtained for the central and lateral incisors. The variation in the Young’s moduli (E_1_, E_2_) of the central and lateral incisors and the ultimate strain ε_12_ of the PDL values at various time points were analyzed. Analysis of variance *P *< 0.05 indicated a statistical significance of the experimental data.

## Results

**Fig. 5:**
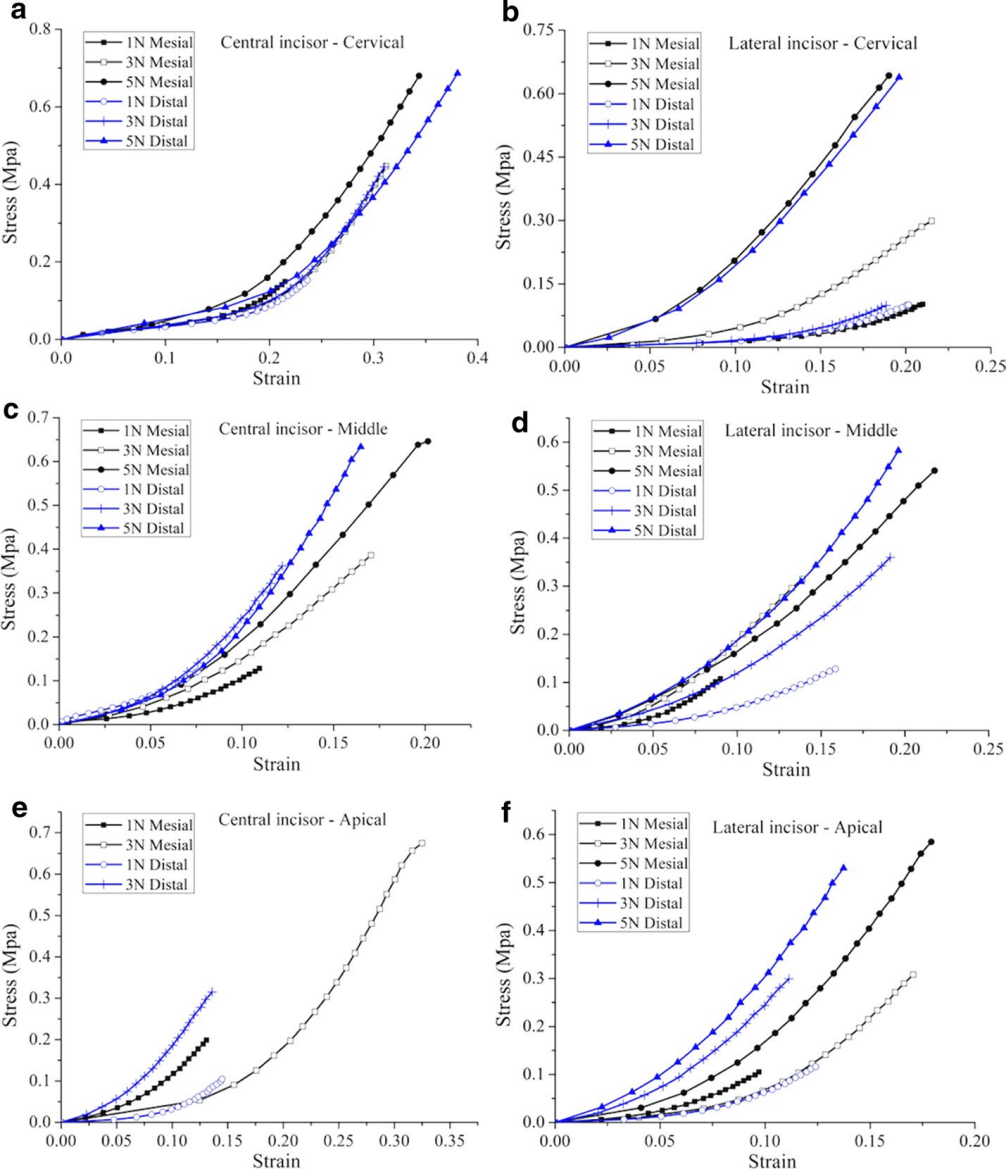
Stress–strain curves of the PDL at different loading rates. **a** Stress–strain curves of central incisor at cervical level; **b** stress–strain curves of lateral incisor at cervical level; **c** stress–strain curves of central incisor at middle level; **d** stress–strain curves of lateral incisor at middle level; **e** stress–strain curves of central incisor at root level; **f** stress–strain curves of lateral incisor at root level

Figure 4b shows the stress–strain curves of the periodontal ligaments at 3 N loading rate as an example, and Fig. 4c shows complete four cycles of loading-hold-unloading curve. In the bilinear elastic model, a low Young’s modulus E_1_ in the first elastic region and a higher one E_2_ in the second region [24] was determined by fitting the stress–strain curve from experiment as shown in Fig. 4b. Both linear sections were separated by a certain ultimate strain ɛ_12_ [25]. Figure 5 shows the PDL stress–strain curves for different loading rates.

**Fig. 6:**
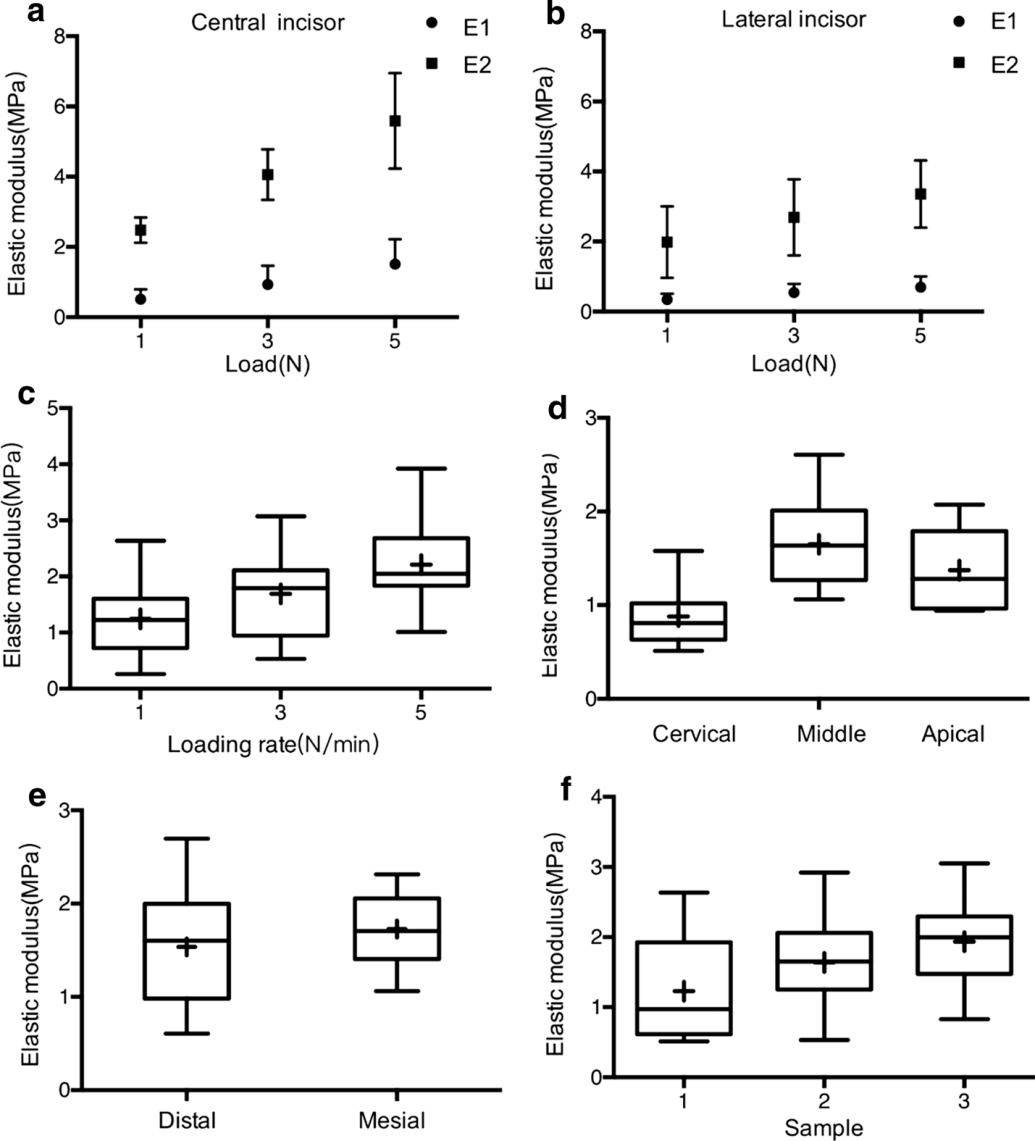
Elastic modulus of the PDL. **a** E_1_ and E_2_ of central incisors; **b** E_1_ and E_2_ of lateral incisors; **c** average elastic modulus at different loading rates; **d** comparison of average elastic moduli among different root levels; **e** comparison of average elastic modulus between medial and distal directions; **f** comparison of average elastic modulus among the three samples. + symbols and vertical bars represent the means and standard deviations of the specimens’ values

Statistically significant differences were detected among the 1, 3, and 5 N loading levels for the different teeth (central and lateral incisors), as shown in Fig. 6a–c. For the central and lateral incisors, the elastic moduli (E_1_ and E_2_) increased with the loading level, as shown in Table 2.

**Table 2:** Elastic moduli (MPa) for central and lateral incisors

Load	Tooth	E_1_	E_2_	Mean ultimate strain ɛ
0–1 N	Central incisors	0.67 ± 0.45	2.63 ± 0.51	0.083
	Lateral incisors	0.33 ± 0.20	2.32 ± 1.15	0.081
0–3 N	Central incisors	1.16 ± 0.73	4.50 ± 1.01	0.103
	Lateral incisors	0.52 ± 0.3	2.75 ± 1.16	0.135
0–5 N	Central incisors	1.82 ± 1.07	5.59 ± 1.23	0.11
	Lateral incisors	0.81 ± 0.36	4.14 ± 1.31	0.167

In addition, the average elastic moduli ${\bar{\text{E}}}$ of E_1_ and E_2_ was calculated to compare the elastic behavior of PDL from different samples. The elastic moduli of the central incisors were greater than those of the lateral incisors (ANOVA, *P* = 0.010, *P *> 0.05), as shown in Fig. 6a, b. Significant differences were detected in the human incisors’ PDLs in relation to different long axial planes (ANOVA, *P* = 0.022, *P* < 0.05). The elastic moduli at the middle planes were greater than at the cervical and apical planes, as shown in Fig. 6d. However, at the cervical, middle, and apical planes, the elastic moduli of the mesial and distal site were not significantly different (ANOVA, *P* = 0.804, *P* > 0.05) (Fig. 6e). Significant differences were detected among the three samples (Fig. 6f).

## Discussion

The mechanical behavior of the human PDL was examined in this study to facilitate better understanding of human tooth mobility. This study was conducted to compare the tensile mechanical properties of the human PDL of central and lateral incisors. Human incisor teeth, including the central and lateral incisors, are all single-root teeth and are similar in their histology but differ in their size and root length. Studying these two types of incisors could avoid the interference factor that arises from root morphological discrepancies.

The mechanical behavior of the PDL play a key role in orthodontics and is not yet sufficiently studied [26]. Figure 5 shows the PDL stress–strain curves for different loading rates. As the strain in the specimen increases, the stress increased in a nonlinear manner. In Fig. 4b, each stress–strain curve is approximated by a bilinear model. In this study, the corresponding to elastic moduli E_1_ and E_2_ were calculated, and the ultimate strain ɛ_12_ was obtained from the stress–strain curve.

As reported in previous studies, the elastic modulus of the PDL varies from 0.01 to 1750 MPa [27]. In this study, the tensile mechanical strength of the human PDL ranged from 0.33 to 6.82 MPa in the incisal region, which is consistent with the results of previous studies [28–30]. The significant change of the slope of the stress–strain curve at the ultimate strain [31] may indicate that, during orthodontic treatment, it is feasible to achieve an effective strain of the PDL before reaching the ultimate strain point. In other words, the use of a small force in orthodontic treatment may be more effective and sufficient. The complex mechanical behavior observed in all connective tissues is directly related to their complex structure and specific components. Collagen fibers comprise more than 50% of the PDL by weight. The major role of the collagen fiber component in the mechanical response of the PDL has been demonstrated in previous research [6, 32]. At the beginning of the stretching process, the collagen fibers are arranged at widely varying angles [5]. During testing, the curly collagen fibers were straightened into a gentle line and then stretched toward the tension axis in a steep line. This may explain the dramatical change of the elastic modulus near the ultimate strain. The experimentally observed deformation behavior of the PDL indicates that the collagen fibers play a dominant role in the PDL’s mechanical response.

In addition to collagen fibers, the PDL also contains blood vessels and nerve endings that are embedded in an amorphous mucopolysaccharide matrix [33] that is responsible for the viscoelastic behavior of the PDL, which help explain the tooth support function of the tissue [7]. When a tooth is subjected to an excessive force, the PDL may dissipate the strain energy stored in the tissues to some extent.

The accurate description of the PDL in different areas is essential for the reasonable prediction of instantaneous tooth movements [27]. A comparison of the elastic moduli at three different loading rates (1 N/min, 3 N/min, and 5 N/min) show that the modulus increases with increasing loading rate, which indicates the viscoelastic response of the PDL. Furthermore, the collagen fibers orientation and the local volume fraction of different constituents result in the observed differences in the mechanical response of the PDL. However, an extensive morphological analysis of the tested specimens would be necessary to quantify the relationships involved.

For the PDL of the different teeth and root levels, the elastic moduli of the mesial and distal sides were not significantly different. However, as Fig. 6a, b show, the elastic moduli (E1 and E2) of the lateral incisors are lower than those of the corresponding area of the central incisors. This indicates that the central incisors can bear larger forces than the same area of the lateral incisor. When restoring teeth using partial dentures, the superficial area of the abutments’ PDL is one of the clinician’s first considerations. Clinicians may also need to consider the ability of the PDL to bear the dental forces of different teeth. Because each tooth root was cut into three slices—cervical, middle, and apical—of the same thickness, three distinct elastic moduli were obtained. The modulus of the middle part was the highest, followed by that of the apical part and then that of the cervical part. As a tooth translates under dental forces, a relatively uniform stress distribution along the axis of the root occurs. However, the PDL’s deformation of different parts could vary from the cervical to the apical because of the differences in the elastic moduli and PDL widths. In this study, statistically significance differences were detected among the tooth samples obtained from different people (ANOVA, *P* = 0.048, *P* < 0.05). This may be the result of individual variation, so a larger sample size should be studied.

This experimental work provides the uniaxial tensile data of the human PDL for the development and validation of the constitutive model of PDL, which play a key role in the finite element simulation of orthodontic tooth movements. However, the PDL is subjected to multiaxial loading during orthodontic treatment. Therefore, experimental studies on the deformation behavior of the PDL in multiaxial stress states can shed light on the deformation behavior under more realistic loading condition during orthodontic treatments.

In the present work, we studied the human incisor teeth, including the central and lateral incisors, which are all single-root teeth and similar in their histology but differ in their size and root length. Studying these two types of incisors can avoid the interference factor that arises from root morphological discrepancies. There are only a few studies on the mechanical behavior of the PDL from molars, since it is extremely difficult to fabricate samples from multi-root tooth due to the small size of molar root. The experimental characterization of the viscoelastic behavior of the PDL from molars will contribute to improving the prediction of orthodontic tooth movement of molars.

## Conclusions

In the uniaxial tensile test of the human PDL, the mechanical behavior of the human PDL of the maxillary central and lateral incisors is studied. The elastic modulus of the human PDL ranged from 0.33 to 6.82 MPa in the incisal region during tensile testing. The elastic modulus of the PDL is probably related to the tooth type, loading rate, and root level.
